# Management Practices and Quality of Care: Evidence from the Private Health Care Sector in Tanzania

**DOI:** 10.1093/ej/uead075

**Published:** 2023-09-18

**Authors:** Timothy Powell-Jackson, Jessica J C King, Christina Makungu, Matthew Quaife, Catherine Goodman

**Affiliations:** London School of Hygiene and Tropical Medicine, UK; London School of Hygiene and Tropical Medicine, UK; Ifakara Health Institute, Tanzania; London School of Hygiene and Tropical Medicine, UK; London School of Hygiene and Tropical Medicine, UK

## Abstract

We measure the adoption of management practices in over 220 private for-profit and non-profit health facilities in 64 districts across Tanzania and link these data to process quality-of-care metrics, assessed using undercover standardised patients and clinical observations. We find that better managed health facilities are more likely to provide correct treatment in accordance with national treatment guidelines, adhere to a checklist of essential questions and examinations, and comply with infection prevention and control practices. Moving from the 10th to the 90th percentile in the management practice score is associated with a 48% increase in correct treatment. We then leverage a large-scale field experiment of an internationally recognised management support intervention in which health facilities are assessed against comprehensive standards, given an individually tailored quality improvement plan and supported through training and mentoring visits. We find zero to small effects on management scores, suggesting that improving management practices in this setting may be challenging.

Over the past two decades, low- and middle-income countries (LMICs) have achieved enormous success in expanding access to health care (Victora *et al*., 2016). Increases in health service coverage have prevented premature death for millions of people (Murray *et al*., 2014). Yet these gains could have been greater were it not for inadequate quality of care. There is extensive evidence that quality of care in most countries is suboptimal, with recent estimates suggesting that it accounts for between 5.7 and 8.4 million deaths each year in LMICs (Kruk *et al*., 2018; National Academies of Sciences, 2018; WHO *et al*., 2018).

Much of the evidence on strategies to improve quality of care have a clinical focus and are targeted at individual health providers (Peabody *et al*., 2006; Rowe *et al*., 2018).^1^ By contrast, less attention has been given to organisation-level factors such as management.^2^ Studies in the United States and UK have shown a strong positive relationship between hospital performance and management (Bloom *et al*., 2015; Tsai *et al*., 2015; McConnell *et al*., 2016).^3^ However, there is little evidence on whether the management-quality relationship holds in lower-income settings. If indeed management does appear to matter, it raises the question of how management practices in hospitals and clinics can be improved. Recent studies provide experimental evidence on the effect of management interventions, but they tend to be outside the health sector (Bloom *et al*., 2013; Bruhn *et al*., 2018; Azulai *et al*., 2020; Muralidharan and Singh, 2020; Iacovone *et al*., 2022).

In this article we assemble a novel data set on management practices and quality of care to investigate this relationship in a sample of small and medium-sized private facilities in Tanzania. We focus on clinical quality because it is the most important dimension of firm performance in the health care sector and its effect on consumer well-being can be very great indeed. There are several reasons to think that the quality-management relationship may not hold in our setting. Formal management practices are less likely to be needed in small and medium-sized firms (McKenzie and Woodruff, 2017). In health systems lacking accountability (e.g., limited enforcement of regulation and absence of clinical audits), health facilities may have greater scope to provide unnecessary care in the pursuit of profit, complicating the relationship between management and clinical quality. Finally, the extent to which adopting management practices divert clinicians away from treating patients is possibly greater where human resource constraints are particularly acute.

To investigate the link between management and quality in our setting, we overcome two major data constraints. First, we address the challenge of measuring the quality of care received by patients when provider behaviour is typically unobserved and the underlying condition of the patient is unknown.^4^ Drawing on Das *et al*. (2016), we used standardised patients (SPs)—healthy people who are trained for two weeks to pose as real patients—who presented with symptoms of asthma, non-malarial febrile illness, tuberculosis and upper respiratory tract infection. We compiled rich data on the history questions, examinations and diagnostic tests completed by the provider, the treatment given to the patient and prices charged. Because we designed the SP cases and hence know the underlying condition presented, we can benchmark the care given against national treatment guidelines to develop condition-specific metrics of process quality. These include correct treatment that we regard as our primary measure of quality of care. We complement the data from SPs with clinical observations of provider-patient interactions to measure compliance with infection prevention and control (IPC) practices. Such practices are vital for health worker and patient safety, particularly in the prevention of health care associated infections (Jha, 2008). The second challenge we address is the measurement of management. Building on the seminal work of Bloom and Van Reenen (2007), we developed a tool with the aim of measuring management in small to medium-sized health facilities in a low-income setting. There is considerable variation in the management score. Over 70% of health facilities adopt less than half of the management practices and about 30% adopt less than one-quarter of the practices.

We find that health facilities that adopt more management practices are associated with higher rates of correct treatment, better adherence to a checklist of history taking questions and examinations, and improved antibiotic prescribing behaviour. These findings are particularly compelling in light of the fact that the SPs allow us to compare quality across providers without confounding due to patient factors since unobserved attributes of the patient are, by design, held constant.^5^ The findings are not sensitive to a wide range of controls, including facility characteristics, geographical dummies and noise dummies related to the survey method. Moreover, estimates are only slightly attenuated when we control for provider qualifications, implying that the presence of better skilled staff is not driving the relationship. We also find a robust positive association between management and compliance with IPC practices. The pattern of results is similar when we use a complementary 55-item management score that captures a distinct set of more process-orientated practices based on data collected independently of ours. While the magnitude of the quality-management relationship varies by outcome, it is large for correct treatment. Going from the 10th percentile to the 90th percentile in the management practice score is associated with a 14-percentage-point or 48% increase in correct treatment. The magnitude is similar to what has been reported in studies examining differences in provider qualification (Das *et al*., 2012) and provider incentives (Das *et al*., 2016). By contrast, the association between management and compliance with IPC practices is small. Nonetheless, the fact that we see improvements in two fundamentally different measures of quality suggests that better management has potential to act on multiple domains of clinical performance.

These findings naturally raise the question of whether there is scope for improving management practices. We leverage a field experiment of a ‘management support’ intervention called SafeCare that provided quality improvement and business expertise in the form of technical assistance to support health facilities through a stepwise certification process, designed by PharmAccess Foundation, an international NGO with an established reputation in the field.^6^ It was developed 12 years ago and refined over the course of implementation in five African countries. A key feature of the intervention is the SafeCare standards—a comprehensive set of criteria covering clinical services and management processes that have been adapted from hospital accreditation standards used in high-income countries. The components of the intervention and its underlying theory of change are strikingly similar to a flagship school management intervention implemented at scale in India (Muralidharan and Singh, 2020). We find that the intervention had no significant effect on our main measure of management practices, with confidence intervals tight around zero. There is a small effect of 4.5 percentage points (10%) on the 55-item measure of management that was constructed from indicators of practices that the intervention was specifically designed to address. Taken together, the findings show that, while it is possible to improve some targeted processes, the intervention did not improve broader management practices. They also go some way to explaining why the intervention did not succeed in improving quality of clinical care, as reported previously in King *et al*. (2021), and suggest that it may have lacked the intensity of successful management interventions.

Our paper contributes to several bodies of empirical literature. Outside of health, the productivity-management relationship is well established (Bloom and Van Reenen, 2007; Bloom *et al*., 2016; 2019). Our article builds on previous empirical work that has shown a positive relationship between hospital management and quality in the UK and the United States (Bloom *et al*., 2015; Tsai *et al*., 2015). We extend this research to small and medium-sized health facilities in a low-income setting, measuring quality with a rich set of process-of-care outcomes that are closely tied to provider performance. There is a body of literature on health care management in LMICs (WHO, 2007; Bradley *et al*., 2012; 2015; Kebede *et al*., 2012; Mabuchi *et al*., 2018). Some articles are simply advocating for the importance of management, while others are based on case study approaches or small sample sizes, making the findings hard to generalise. We contribute to a small number of field experiments of management interventions. While those in private firms show positive effects on business outcomes (Bloom *et al*., 2013; Bruhn *et al*., 2018; Iacovone *et al*., 2022), other studies in schools, health facilities and the civil service suggest that it is difficult to improve managerial quality (Azulai *et al*., 2020; Muralidharan and Singh, 2020; Dunsch *et al*., 2023).^7^

The rest of the article is organised as follows. Section 1 presents the context and the data used in the paper. Section 2 presents the empirical approach and results examining the relationship between management and quality of care. Section 3 estimates the effect of the SafeCare intervention on management practices. Section 4 concludes.

## 1. Context and Data

### 1.1. Health Care in Tanzania

Health care in Tanzania is delivered by both public and private hospitals and clinics. The private sector includes both not-for-profit and for-profit facilities, accounting for 25% of facilities nationally, with the vast majority being small and medium-sized (Darcy *et al*., 2019).^8^ Such facilities treat patients with a wide range of infectious diseases of public health importance, such as malaria, diarrhoea and sexually transmitted diseases, as well as non-communicable conditions such as diabetes, hypertension and asthma. Non-profit facilities tend to be faith based and have long been important providers of primary and secondary care, particularly in rural areas (Mackintosh *et al*., 2016). Private providers are extremely heterogeneous, ranging from small clinics to international-standard hospitals.

It is commonly understood that the quality and safety of care in much of the private sector is inadequate. In LMICs more broadly, private providers perform better on timeliness and hospitality to patients than public facilities, but more frequently violate medical standards and are more likely to provide unnecessary testing and treatment (Berendes *et al*., 2011). Small and medium-sized private facilities can be considered at particular risk of quality and safety problems. They lack the higher-level supervision and more developed organisational structures of large hospitals, and staff may be inappropriately qualified, or have limited access to continuing medical education. Moreover, most for-profit facility owners work in their facilities and are paid predominately fee-for-service, generating high-powered financial incentives. Facility staff may also lack business and management skills, and struggle to access funds to invest in improved quality and safety. Regulation of small and medium-sized facilities is often under-developed and out-dated (World Bank, 2011; Sheikh *et al*., 2015).

### 1.2. SafeCare Intervention

Our study on management relies on data collected in the context of a randomised controlled trial of the SafeCare programme. The intervention was developed in 2011 by PharmAccess Foundation, an established International NGO. As of 2019, SafeCare had been implemented in almost 2,000 health facilities in five African countries. PharmAccess has used this experience to refine SafeCare over the years and develop an increasingly more sophisticated data platform. The intervention centres around the SafeCare standards—a comprehensive set of criteria covering clinical services and management processes that have been adapted from hospital accreditation standards used in high-income countries. The SafeCare standards were accredited by the International Society for Quality in Health Care in 2017.

The intervention provided technical assistance to support health facility owners and managers in adopting the SafeCare standards. Underpinning the intervention was an extensive database documenting health facility performance on a precisely defined set of indicators corresponding to these standards. The intervention comprised four main components.

- *SafeCare assessment*. Health facilities were visited by trained assessors who administered an assessment covering 170 basic standards. Facilities were given a report of the assessment that highlighted the level of performance (a certificate indicating the level on a rating of one to five) and provided an analysis of gaps. Follow-up assessments were carried out 18 to 24 months later to evaluate improvements in the adoption of standards.
- *Quality improvement plan*. Based on an analysis of the SafeCare assessment, quality advisors worked with the facility management to develop a quality improvement plan that prioritised actions and resources for the adoption of additional standards.
- *Training*. To support facilities in adopting standards, staff were given on-site and classroom training on topics such waste management, record keeping, customer care and marketing.
- *Mentoring visits*. To further support staff, facilities were given regular progress monitoring visits by the quality advisors who assessed progress against the quality improvement plan.^9^

The trial of the SafeCare intervention allocated facilities to treatment or control. Treatment facilities were exposed to the full package of activities, as described above. Control facilities were also given SafeCare assessments at both baseline and endline since these data were used as part of the evaluation and received a report of the initial assessment, handed to them with no further feedback. Baseline data collection and enrolment into the trial was staggered for logistical reasons over the course of 2016. The endline assessment was conducted by the research team almost two years after the baseline assessment.^10^

### 1.3. Data Collection

#### 1.3.1. Health facility sample

Our sample comprises private health care facilities that participated in the trial of SafeCare. It includes dispensaries, health centres and hospitals in both the private non-profit and for-profit sectors. We excluded referral hospitals as well as those providing only specialist services, such as mental health care. We recruited 237 facilities at baseline. During the course of the study, nine facilities closed down, providing us with a sample of 228 health facilities. The characteristics of the sample of health facilities are shown in Table 1. By design, approximately half the sample is for-profit while the remainder comprises non-profit faith-based facilities. The majority of facilities are dispensaries (55%), while 29% are health centres and 16% hospitals. Around four-fifths are located outside of the largest city, Dar es Salaam, and 40% are in rural areas.^11^

**Table 1.** Summary Statistics on Facility Characteristics and Quality-of-Care Metrics.

Variable	Mean	SD	Min	Max	Facilities	Obs
*Panel A: management*						
Management score, facility survey (0 to 1 scale)	0.38	0.18	0	0.90	228	228
Management score, SafeCare assessment (0 to 1 scale)	0.46	0.15	0.06	0.91	221	221
*Panel B: quality of care and other outcomes*						
Correct treatment	0.28	0.45	0	1	227	909
Proportion of checklist items completed	0.32	0.14	0	0.83	227	908
Unnecessary care	0.82	0.39	0	1	227	909
Number of antibiotics prescribed	0.69	0.59	0	3	227	909
Prices charged to standardised patients (US$)	4.80	3.53	0	23.20	227	909
Infection prevention and control compliance	0.56	0.50	0	1	220	29,608
*Panel C: covariates*						
SafeCare intervention group						
Treatment	0.49	0.50	0	1	228	228
Control	0.51	0.50	0	1	228	228
Type of umbrella organisation						
APHFTA	0.49	0.50	0	1	228	228
CSSC	0.51	0.50	0	1	228	228
Facility type						
Dispensary	0.55	0.50	0	1	228	228
Health centre	0.29	0.45	0	1	228	228
Hospital	0.16	0.37	0	1	228	228
Facility location						
Inside Dar Es Salaam	0.18	0.39	0	1	228	228
Outside Dar Es Salaam	0.82	0.39	0	1	228	228
Location type						
Urban	0.31	0.46	0	1	228	228
Peri-urban	0.27	0.44	0	1	228	228
Rural	0.42	0.49	0	1	228	228

*Notes*: The table provides summary statistics on key variables from multiple data sources, including the health facility survey, standardised patient survey, clinical observations, SafeCare assessment survey and a census of health facilities. APHFTA is the Association of Private Health Facilities in Tanzania that represents mainly for-profit facilities; CSSC is the Christian Social Services Commission that represents most mission facilities.

#### 1.3.2. Measuring management

Our main measure of facility management practices is based on a tool we developed, motivated by the work of Bloom and Van Reenan (2007); Bloom *et al*. (2015). We asked 13 management questions across four domains: operations; performance monitoring and targets; human resource management and financial management (Online Appendix Table A1). We reviewed the hospital version of the World Management Survey (Bloom *et al*., 2015) and other relevant tools such as the Management and Organizational Practices Survey (McKenzie and Woodruff, 2017; Bloom *et al*., 2019), and adapted practices to our study context. We focused on basic practices for which there is a consensus that small and medium-sized facilities in a low-income country setting would benefit by adopting them. We originally designed a set of closed-ended questions to be asked through face-to-face interview with managers. However, pilot testing raised concerns about the reliability of these self-reports. We therefore decided to verify responses through observation of the practice itself or documentation providing evidence of the practice, and it is these data we use to construct our measure of management.

**Fig. 1.**
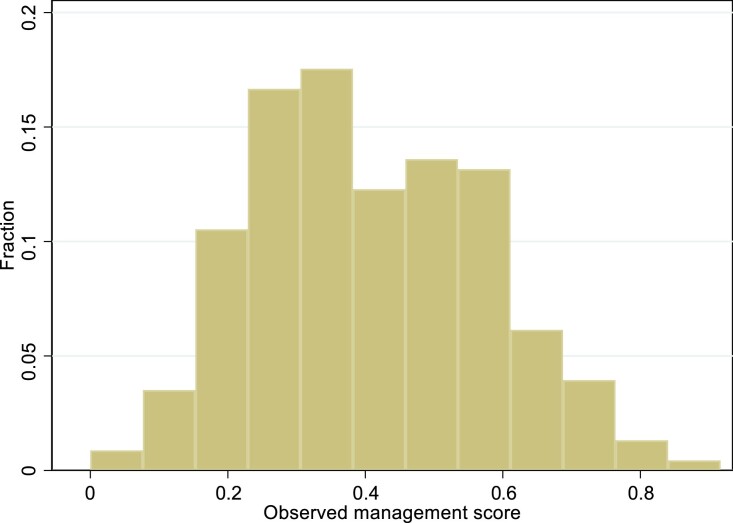
Distribution of the Facility Survey Management Score.

We aggregate the results from the 13 management questions into a single metric that we refer to as the ‘health facility survey management score’. Responses to each question are first scored on a 0 to 1 scale, with the best response receiving 1 and the worst response 0. For example, in response to the question ‘do you use patient records?’, the category ‘patient records for all patients’ is assigned a value of 1, ‘patient records for some patients’ is given 0.5 and ‘no patient records system’ is given 0. We then take the unweighted average of the scores from the 13 questions to generate a summary score. This score ranges from 0 to 1, and is interpreted as the proportion of the maximum score obtainable.^12^ There is a wide dispersion in the score; the range is 0 to 0.92 and the IQR is 0.23–0.53 (Figure 1). The mean score is a relatively low 0.38, indicating that facilities on average adopt 38% of the practices (Table 1). Over 70% of health facilities adopt less than half of the management practices measured, suggesting that a majority of health facilities are poorly managed. Just over 30% of health facilities adopt less than one-quarter of the management practices.

We construct a second complementary measure of management, using an independent source of data collected as part of the SafeCare intervention. The SafeCare assessment survey measured compliance with a set of ‘criteria’ based on international hospital accreditation standards that have been adapted for use in low-resource settings. The tool covers 170 practices regarded as essential for a well-functioning health facility.^13^ The assessment involved a one- to two-day on-site visit in which facility staff were interviewed, records were reviewed and practices were observed. We make use of the data from the survey carried out in 2018, around the same time as our health facility survey. Prior to data collection, we pre-specified 55 practices included in the assessment survey that we considered to be management related (Online Appendix Table A2). These procedural activities are distinct from the management practices we measured: they have their origins in hospital accreditation, are less grounded in the academic management literature and are more process orientated, giving particular emphasis to patient safety. They are nevertheless of interest, not least because they were specifically targeted by the intervention. As with our preferred measure of management, this score is scaled from 0 to 1 and is interpreted as the proportion of management practices adopted (see Online Appendix A). The score has a mean of 0.46 and is widely dispersed (range is 0.06 to 0.91 and the IQR is 0.35 to 0.57). The correlation with the health facility survey management score is positive, but substantially less than, 1 (*r* = 0.54, *p*-value < 0.001), suggesting that it is indeed capturing different aspects of managerial performance.

#### 1.3.3. Quality-of-care metrics

Quality of care is a multidimensional construct (Institute of Medicine (US) Committee on Quality of Health Care in America, 2001). We focus on ‘process-of-care’ measures (Donabedian, 2005) because they are informative about the care actually received by patients and are the most direct measure of provider behaviour. The study of processes of care is also motivated by the idea that health facilities have more control over them than health outcomes such as survival. Nevertheless, processes of care are challenging to measure because they concern the private interaction between clinician and patient. We employed two complementary methods that measured different dimensions of process quality: standardised patients to measure adherence to established clinical guidelines and clinical observations to measure compliance with IPC practices (see Online Appendix A for a detailed description of the data).

##### Standardised patients

Standardised patients are healthy people, who covertly pose as real patients and respond to the clinician's actions as a real patient would. Increasingly they are being used to evaluate the quality of clinical care, particularly in settings where routine data are not available through medical records (Kwan *et al*., 2019). SPs are trained to portray a precise set of symptoms and consistently follow a script that guides them in how to respond to questions the clinician may have during history taking. We developed four SP cases—asthma, non-malarial febrile illness, tuberculosis and upper respiratory tract infection—adapting protocols and scripts used in previous studies (Das *et al*., 2012; 2016; Mohanan *et al*., 2015). Health facilities were told that an SP would be visiting their facility unannounced at some point over the next three months, but they were given no further details. We sent the four SP cases to each health facility in the sample, randomly allocating fieldworkers to health facilities within each region.^14^

SP interactions provide information to measure a rich set of quality of clinical care outcomes. Our main outcome is correct treatment (see Online Appendix Table A4), which measures whether the SP received care consistent with the national treatment guidelines (Ministry of Health, 2017). In the case of the asthma SP, for example, treatment was defined as correct if the SP was prescribed an inhaled bronchodilator or steroid. We captured the following additional measures of quality: adherence to an essential checklist of questions and examinations, provision of unnecessary care and the number of antibiotics prescribed. Checklist adherence was measured both as a percentage of condition-specific checklist items completed and as an index generated using item response theory (IRT) to give more weight to items that discriminate better among providers. Unnecessary care was defined as drugs prescribed for which there was no evidence of treatment effectiveness or symptomatic relief, and inappropriate tests ordered. Antibiotics were not indicated for any of the SP cases and therefore reflect inappropriate prescribing behaviour.^15^

Table 1 presents summary statistics on the SP quality metrics. Correct treatment was 28%, such that health providers followed treatment guidelines in less than one-third of SP visits. There was, however, substantial variation in correct treatment across conditions, ranging from 6% for asthma to 72% for non-malarial febrile illness. Adherence to the essential checklist of questions and examinations was on average 32%, with little variation across SP cases (result not shown). Unnecessary care was prevalent, with SPs in 81% of visits receiving medications or tests classified as unnecessary. Health providers prescribed antibiotics at an average rate of 0.69 drugs per visit, suggesting that overprescribing is a major issue in this setting.

##### Clinical observations

We adapted previous tools (WHO, 2008; 2009; Bedoya *et al*., 2017) for clinical observations to measure health provider compliance with IPC practices. The assessment was based on the concept of indications—that is, moments in a provider-patient interaction that present an infection risk to either patient, provider or both. For example, if the provider takes the patient's temperature with a thermometer, a patient is exposed to an infection risk. For every indication, there is a corresponding action. In the case of a thermometer, a corresponding action is disinfecting the equipment before and after use with rubbing alcohol or bleach. Compliance means the correct action was taken in response to an indication. The tool specified 21 indications and corresponding actions across the following five domains of IPC: hand hygiene, glove use, injection and blood draw safety, disinfection of reusable equipment and waste management (Online Appendix Table A6).^16^ Fieldworkers observed provider-patient interactions in consultation rooms, laboratories and injection or dressing rooms. In addition to IPC compliance, they collected information on the characteristics of each patient and the qualifications of each provider in attendance. Table 1 presents summary statistics on compliance with IPC practices. Overall compliance was 56%. There was wide variation in compliance by domain of IPC (data not shown). Compliance was extremely poor for disinfection of reusable equipment (5%) and hand hygiene (8%) and almost universal for injections and blood draw (96%), suggesting some behaviours are ingrained into routine practice while others are seemingly difficult to establish.

## 2. Management and Quality of Care

In this section, we explore whether facility management practices are associated with quality of clinical care. Although there is evidence from some high-income countries of an association between hospital management practices and the quality of clinical care, this has not been established in any LMIC.

### 2.1. Estimation Framework

We estimate the relationship between management practices and quality, where the latter is measured by correct case management of SPs and compliance with IPC practices. For SPs, we estimate


(1) \begin{eqnarray*}
{q}_{( {i( {scf} )r} )} = {\beta }_0 + {\beta }_1\textit{MNGM{T}}_f + \beta _2^{\mathrm{^{\prime}}}{X}_f + {\delta }_s + {\delta }_c + {\delta }_r + {\mu }_{( {i( {scf} )r} )},
\end{eqnarray*}

which regresses quality *q* (correct treatment and other SP measures of quality) in consultation *i* with standardised patient *s* presenting case *c* in health facility *f* and region *r* on the management practice score. The coefficient of interest is ${\beta }_1$. Our analysis considers both measures of management: the facility survey management score and the management score from the SafeCare assessment. We control for ${X}_f$, a vector of facility characteristics: the type of health facility, whether it is for-profit or non-profit, urban location and the SafeCare trial arm. Although we explore different specifications, our main model includes fixed effects for SPs $({\delta }_s)$, case $( {{\delta }_c} )$ and region $({\delta }_r)$ to account for systematic differences across them. Given that the analysis is at the level of the SP visit and we have multiple visits per facility, we cluster SEs at the facility level. The regression implicitly weights each facility equally because facilities received the same number of SPs.

Although we lack a natural experiment to create variation in management, we are careful to establish the robustness of the relationship. Our use of SPs means that we can compare quality across providers without confounding due to case mix and other patient characteristics since unobserved attributes of the patient are, by design, held constant (Das *et al*., 2016). This guards against an important source of endogeneity that afflicts studies relying on measures of quality derived from real patients.^17^ If, for example, harder-to-treat patients sort to better managed facilities, quality of care may appear worse than is actually the case, attenuating the relationship between management and quality of clinical care.^18^ We also gauge the threat from omitted variable bias by presenting bounds on coefficient estimates under the assumption of equal selection on observables and unobservables (Oster, 2019). We set ${R}_{max}$ equal to $1.3\ \tilde{R}$, where $\tilde{R}$ is the *R*^2^ of the full control model. This approach helps us to get a handle on the importance of potential confounders for which we have no data, such as the quality of leadership and organisational culture. Finally, we control for the qualifications of clinical staff, to address the concern that the correlation could be driven by the fact that better managed facilities have more skilled health workers. This in itself does not preclude a role for management—better managed facilities may be able to attract better talent. It does imply, however, that if the supply of skilled staff is inelastic, improving management in one facility will lead to quality declines in others, as the best clinicians are poached. We provide interpretation of these results later.^19^

Using the clinical observation data, we estimate


\begin{eqnarray*}
{q}_{( {n( {ipf} )r} )} = {\beta }_0 + {\beta }_1\textit{MNGM{T}}_f + \beta _2^{\mathrm{^{\prime}}}{X}_f + \beta _3^{\mathrm{^{\prime}}}{Z}_i + {\delta }_n + {\delta }_r + {\mu }_{( {n( {ipf} )r} )},
\end{eqnarray*}

regressing compliance with IPC practices *q* for indication *n* in patient observation *i* with health provider *p* in health facility *f* and region *r* on the management practice score; ${\beta }_1$ is again the coefficient of interest. With clinical observations, we lose the advantage of SPs to be able to compare across providers without confounding due to patient case mix. At the same time, IPC compliance, as a measure of patient safety, is less likely to be conditional on the patient. Nonetheless, we adjust for patient characteristics, ${Z}_i$, by including controls for patient age and gender. Given that compliance varies substantially by IPC domain, we include indication fixed effects, ${\delta }_n$. As before, we cluster SEs at the facility level. It bears noting that we observed patients for a fixed number of hours, which means that facilities with higher patient volume will implicitly be given a greater weight in the analysis.

### 2.2. Results

Table 2 presents our main results from the SPs. Panel A shows results from regressions in which the independent variable of interest is the primary measure of management from our health facility survey. Including no controls, we find a statistically significant and positive relationship between management practices and correct treatment (column (1)). The coefficient falls slightly to 0.29 when we control for facility characteristics (column (2)) and remains the same with the inclusion of SP and case fixed effects, with results significant at the 1% level (column (3)). When we limit the analysis to the sample of health facilities in the control group of the SafeCare trial, the relationship between management and correct treatment becomes stronger (column (4)). Bias-adjusted point estimates remain quantitatively similar to those from the full control models, under the assumption of equal selection on observables and unobservables. In Online Appendix Table B2, we report estimates by condition. With the exception of asthma, the management-quality relationship is positive and statistically significant, with the coefficient of management ranging from 0.30 for upper respiratory tract infection to 0.49 for tuberculosis.

**Table 2.** Management and Quality of Care for Standardised Patients.

	(1)	(2)	(3)	(4)	(5)	(6)	(7)	(8)
Sample	Full	Full	Full	Control group	Full	Full	Full	Full
Dependent variable	Correct treatment	Correct treatment	Correct treatment	Correct treatment	Proportion of checklist items completed	IRT checklist score	Any unnecessary care	Number of antibiotics prescribed
*Panel A: facility management survey*								
Management score	0.337***	0.291***	0.290***	0.489***	0.104***	0.740***	−0.036	−0.256*
	(0.069)	(0.073)	(0.073)	(0.108)	(0.038)	(0.221)	(0.078)	(0.138)
General controls	No	Yes	Yes	Yes	Yes	Yes	Yes	Yes
SP and case fixed effects	No	No	Yes	Yes	Yes	Yes	Yes	Yes
Facilities	227	227	227	116	227	227	227	227
Observations	909	909	909	465	908	908	909	909
*R* ^2^	0.018	0.046	0.398	0.441	0.225	0.221	0.157	0.213
Bias-adjusted coefficient	−	−	0.267	0.499	0.084	0.614	−0.013	−0.217
*Panel B: SafeCare standards*								
Management score	0.308***	0.228**	0.213*	0.446**	0.196***	1.264***	−0.140	−0.496**
	(0.088)	(0.107)	(0.110)	(0.180)	(0.049)	(0.257)	(0.102)	(0.194)
General controls	No	Yes	Yes	Yes	Yes	Yes	Yes	Yes
SP and case fixed effects	No	No	Yes	Yes	Yes	Yes	Yes	Yes
Facilities	219	219	219	110	219	219	219	219
Observations	877	877	877	441	876	876	877	877
*R* ^2^	0.011	0.039	0.395	0.444	0.238	0.231	0.160	0.225
Bias-adjusted coefficient	−	−	0.156	0.402	0.209	1.330	−0.166	−0.515

*Notes*: The table reports OLS coefficients with SEs in parentheses, clustered at the facility level. In panel A, the management score from the facility survey is the unweighted average of the score on each of the 13 management questions. In panel B, the management score from the SafeCare assessment is the unweighted average of the score on each of the 55 management-related items that were pre-specified prior to data collection. Observations are at the SP visit level. The full sample is used in all regressions, with the exception of column (4) in which the sample is limited to facilities assigned to the control arm of the SafeCare field experiment. General controls capture characteristics: facility type, region, profit status, urban location and SafeCare trial arm. SP fixed effects are a dummy variable for each of the SP fieldworkers. Case fixed effects are a dummy for each of the SP case presentations. The bias-adjusted coefficient is the value of β produced when $\delta = 1$ and ${R}_{max} = 1.3\tilde{R}$. *** significant at 1%, ** at 5%, * at 10%.

The coefficient in our main specification (column (3)) implies that a 10-percentage-point increase in the management score is associated with a 2.9-percentage-point improvement in correct treatment. To further interpret the magnitude, consider that the management score has an SD of 0.179 such that a one-SD increase in the score is associated with a 5.2-percentage-point increase in correct treatment (or a rise of 19% over the mean of 28%). Alternatively, moving from the 10th percentile to the 90th percentile in the management score, an increase of 0.47 in the score is associated with a 13.6-percentage-point (or 48%) increase in correct treatment. Given the lack of similar studies in the literature, it is not easy to put these magnitudes into perspective. In India, correct treatment was 15 percentage points higher when the doctors were assessed by SPs in their private practice (paid by fee-for-service) than when the same doctors were assessed in their public practice (paid by salary) (Das *et al*., 2016), and 14 percentage points higher when doctors were compared with informal providers in the treatment of tuberculosis (Kwan *et al*., 2018).

The remaining columns in Table 2 show the results for other SP measures of process quality. We find a statistically significant and positive relationship between management practices and checklist adherence. A 10-percentage-point improvement in the management index is associated with 1.0 percentage point more checklist items completed (column (5)) and an increase of 0.07 SDs on the IRT-scaled score (column (6)). There is no evidence of an association between management practices and unnecessary care (column (7)). Finally, the result on antibiotics indicates that management practices are correlated with a reduction in the rate of antibiotic prescriptions (column (8)). These findings lend support to the possibility that the positive relationship between management practices and correct treatment is driven, in part, by greater provider effort. They are also reassuring in the sense that the positive relationship between management and correct treatment does not coexist with better managed facilities providing more unnecessary care. A possible concern could have been that facilities that achieve higher rates of correct treatment do so by simply providing more care—much of which may be unnecessary. In fact, the results show that management practices are associated with less care in one critical area: antibiotic prescribing behaviour. In Online Appendix Table B3, we show that there is no significant association between management and prices charged. Although estimates are imprecise, the suggestion is that the market in Tanzania may not provide a strong incentive for facilities to improve management given that better management is not reflected in substantially higher prices.

As discussed, providers working in better managed facilities may be more skilled. If this reflects the fact that facilities have better qualified providers *because* they are well managed, our estimates in Table 2 capture both the direct and indirect effects of management. However, if provider skill is simply confounding the relationship, our estimates will be biased. In Online Appendix Table B4, we control for the number of medical officers as a proportion of clinical staff in facilities. The magnitudes of the estimates are attenuated slightly (e.g., correct treatment falls to 0.25) but, with the exception of antibiotic prescribing, the findings remain statistically significant. This indicates that the relationship exists independent of provider qualifications and that, even if provider skill is a mediator, it is not a key driver of the relationship.

Panel B of Table 2 shows the results using the management score from the SafeCare assessment. As previously noted, we view this measure of management practices as complementary to our own as it draws on distinct elements from hospital accreditation. The pattern of results is similar to those reported in panel A. In particular, there is a positive and statistically significant association between management practices and correct treatment, with estimates of the same order of magnitude as those on our preferred management score (columns (1)–(4)).

We further examined the robustness of our main finding on correct treatment along a number of dimensions (see Online Appendix Table B6). First, the results are robust to the inclusion of additional facility controls such as the number of beds. Second, we excluded from the analytical sample SP interactions that were ‘detected’ in the call-back survey, and find no change in the results. Third, we used patient volume (number of patient visits per month) to weight the data. By fitting a weighted version of (1), the results reveal the association between management and quality for the average patient. We find that the results remain largely unchanged when we applied the weight. Fourth, we show that the results are not sensitive to a probit regression model. Fifth, in an effort to reduce measurement error, we generate an alternative management score by taking the average of our preferred management score based on observation and one based on responses to identical questions asked during interview. The results are similar. Sixth, we find that other methods of generating a summary management score, namely the primary factor from factor analysis and the *z*-score, do not qualitatively alter the findings.

Table 3 reports results on the relationship between management practices and compliance with IPC practices, using data from the clinical observations. Focusing first on the measure of management from our survey in panel A, we find that there is a statistically significant and positive correlation between management practices and compliance in the absence of controls (column (1)). When we include general controls and patient controls in columns (2) and (3), respectively, the positive relationship remains. The estimate in our preferred specification indicates that a 10-percentage-point increase in the management score is associated with a 0.62-percentage-point increase in compliance with IPC practices, significant at the 10% level (column (4)). By any measure, this estimate is small in magnitude. For example, moving from the 10th percentile to the 90th percentile in the management score (an increase of 0.47 in the score) is associated with a 2.9-percentage-point increase in compliance. The coefficient estimate is larger when we limit the facility sample to those assigned to the control group (column (5)). Panel B presents the results from regressions with the SafeCare management score. They also show a positive association between management practices and compliance with IPC practices that is mostly (but not always) statistically significant. As with the SPs, we controlled for provider qualifications, except this time with fine-grained information specific to the provider attending each patient observed.^20^ The estimate was slightly attenuated. We performed other robustness checks, with results stable across the different specifications (see Online Appendix Table B4).

**Table 3.** Management and Compliance with Infection Prevention and Control Practices.

	(1)	(2)	(3)	(4)	(5)
Sample	Full	Full	Full	Full	Control group
Dependent variable	IPC compliance	IPC compliance	IPC compliance	IPC compliance	IPC compliance
*Panel A: facility management survey*					
Management score	0.089**	0.113***	0.106***	0.062**	0.088**
	(0.038)	(0.037)	(0.038)	(0.031)	(0.038)
General controls	No	Yes	Yes	Yes	Yes
Patient controls	No	No	Yes	Yes	Yes
Indication fixed effects	No	No	No	Yes	Yes
Facilities	220	220	220	220	220
Observations	29,608	29,608	29,608	29,608	15,242
*R* ^2^	0.001	0.006	0.008	0.640	0.644
Bias-adjusted coefficient	−	−	−	0.047	0.070
*Panel B: SafeCare assessment*					
Management score	0.060	0.144***	0.137***	0.077**	0.071
	(0.042)	(0.050)	(0.050)	(0.037)	(0.054)
General controls	No	Yes	Yes	Yes	Yes
Patient controls	No	No	Yes	Yes	Yes
Indication fixed effects	No	No	No	Yes	Yes
Facilities	212	212	212	212	108
Observations	29,054	29,054	29,054	29,054	14,773
*R* ^2^	0.000	0.006	0.008	0.643	0.651
Bias-adjusted coefficient	−	−	−	0.088	0.041

*Notes*: The table reports OLS coefficients with SEs in parentheses, clustered at the facility level. The dependent variable is compliance with infection prevention and control practices in all regressions. Observations are at the level of IPC indication. In panel A, the management score from the facility survey is the unweighted average of the score on each of the 13 management questions. In panel B, the management score from the SafeCare assessment is the unweighted average of the score on each of the 55 management-related items that were pre-specified prior to data collection. The full sample is used in all regressions, with the exception of column (5) in which the sample is limited to facilities assigned to the control arm of the SafeCare field experiment. General controls capture characteristics: facility type, region, profit status, urban location and SafeCare trial arm. Patient controls are age and gender of the patient observed. Indication fixed effects are a dummy variable for each IPC indication. The bias-adjusted coefficient is the value of β produced when $\delta = 1$ and ${R}_{max} = 1.3\tilde{R}$. *** significant at 1%, ** at 5%.

## 3. Firm-Level Experiment

The findings thus far show that there is a robust association between management and clinical quality. This naturally gives rise to the question of whether there is scope for improving management in health facilities in Tanzania. In this section, we leverage a field experiment of SafeCare to examine whether a management support intervention improved management practices.

### 3.1. Experimental Design and Estimation

The firm-level trial involved randomisation at the level of the health facility.^21^ We randomly assigned health facilities to treatment or control as follows. To ensure experimental balance across relevant characteristics, we generated 14 strata on the basis of geographic zone, whether a health facility was a hospital, and the association that the facility belonged to. We then randomly allocated health facilities to treatment or control with a computer random number generator and an algorithm that stratified the sample so that the proportion allocated to each of the two arms was the same within each stratum. The result is that we have 118 treatment and 119 control facilities.

The recruitment of health facilities and hence the start of the intervention was staggered over time in three phases. Baseline data collection was conducted by assessors, who visited each health facility to administer the SafeCare assessment and collect additional information on facility characteristics. At the end of the visit, once data collection activities had been completed, a sealed envelope was opened to reveal the treatment assignment to mangers at the health facility. Online Appendix Table B8 shows that the treatment and control health facilities were similar across a range of characteristics.

We estimate the intention-to-treat effect of the intervention on our two measures of management. We run OLS regressions of the management score on a binary variable indicating whether the health facility was assigned to the treatment group. We report robust SEs. We estimate one model with stratum fixed effects and a second with additional controls, including baseline characteristics of the facility. For completeness, we also estimate effects on correct treatment and IPC compliance, while noting that they have been reported previously (King *et al*., 2021).^22^

### 3.2. Results

**Fig. 2.**
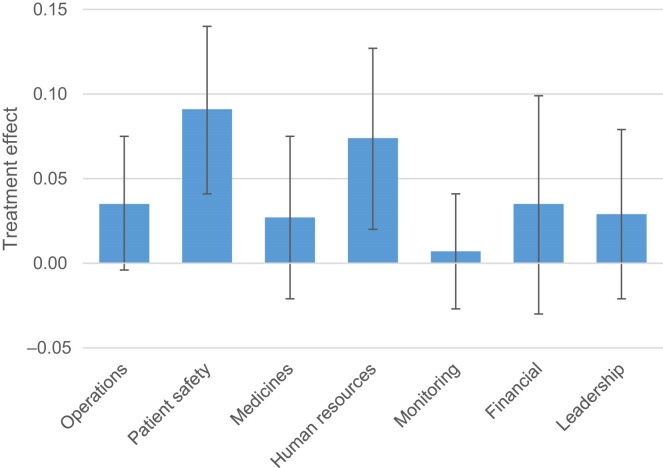
Treatment Effects on Domains within the SafeCare Management Score. *Notes:* The figure shows the effect of the SafeCare intervention on domain-specific management scores. The error bars are 95% CIs based on robust SEs. Treatment effects are based on OLS regressions that include randomisation stratum fixed.

Table 4 shows the effect of the intervention on our facility survey management score and the management score based on the SafeCare assessment. Results show the SafeCare intervention had no significant effect on management practices as measured by the facility survey, with estimates close to zero in both models (column (1)). By contrast, there is a positive and significant effect of the intervention on the SafeCare management score, with estimates ranging from 4.5 to 4.8 percentage points (column (2)).^23^ There was no evidence of an effect of the intervention on correct treatment, as measured using the SPs (column (3)). For IPC compliance, the point estimates are small, suggesting that the intervention had at most a small positive effect of 2.5 percentage points, which is unlikely to be clinically meaningful (column (4)). We further explored the treatment effect on individual domains and items within both the management scores. Across the 13 items that make up the facility survey management score, there was a significant negative effect on two items (both significant at the 5% level). Across the 55 items in the second management score, there was a significant positive effect on 13 items (10 items significant at the 5% level and three items significant at the 10% level). In terms of domains with the SafeCare management score, there was a significant effect on practices related to patient safety and human resource management (Figure 2).

**Table 4.** Experimental Effect of SafeCare on Management Practices and Quality of Care.

	(1)	(2)	(3)	(4)
Dependent variable	Facility survey management score	SafeCare management score	Correct treatment	IPC compliance
*Panel A: stratum fixed effects*				
Treatment	−0.005	0.048***	−0.028	0.018
	(0.022)	(0.018)	(0.024)	(0.013)
Control mean	0.38	0.44	0.29	0.55
Facilities	228	221	227	220
Observations	228	221	909	29,608
*Panel B: stratum fixed effects and further controls*				
Treatment	−0.010	0.045***	−0.029	0.025**
	(0.022)	(0.015)	(0.025)	(0.011)
Control mean	0.38	0.44	0.29	0.55
Facilities	228	221	227	220
Observations	228	221	909	29,608

*Notes*: The independent variable is a treatment dummy for whether the facility was assigned to the intervention arm of the SafeCare trial. The sample and level of observation varies according to the dependent variable. Columns (1) to (2) report OLS coefficients with robust SEs in parentheses. Columns (3) to (4) report OLS coefficients with SEs in parentheses, clustered at the facility level. In panel A, the regressions include stratum fixed effects, a dummy variable for each randomisation stratum. In panel B, the regressions control for stratum fixed effects and the baseline number of consulting rooms and number of beds, as well as the following additional controls: column (1) controls for no additional covariates; column (2) controls for baseline management score; column (3) controls for SP and case fixed effects; column (4) controls for indication fixed effects. *** significant at 1%, ** at 5%.

How should we interpret the results from the two measures of management? The management score based on the SafeCare assessment was constructed from data on the adoption of practices that the intervention was specifically designed to address. The health facility survey management score, on the other hand, was developed independently with its foundations in the economics literature.^24^ It is perhaps unsurprising then that the measure most closely related to the practices targeted by the intervention showed greater improvement. However, two points are worth noting. First, the effect on the SafeCare management score is small in magnitude. The positive effect of 4.5 percentage points is equivalent to an increase of 10% in the score and is considerably smaller than the 25 percentage points achieved in a trial of intensive management consultancy in Indian textile firms (Bloom *et al*., 2013). Second, it seems that the intervention was not able to improve the broader set of management practices that we measured. From this standpoint, both sets of results are consistent in suggesting that supporting health facilities at scale to adopt modern management practices is challenging. It is instructive to note that the management field experiment in India cost approximately ${\$}$288,000 (2018 prices) per firm compared with a little over ${\$}$8,000 (2018 prices) per facility in our trial.^25^

The SafeCare intervention was implemented by dedicated programme staff and was considered well resourced for the setting. Nonetheless, it is plausible that the intensity of the intervention was still low to have a large effect on management. Other possible reasons for the limited impact on management practices include an insufficient follow-up period of two years and the reluctance of staff to attend training sessions without additional financial incentives.^26^ Our findings mirror those from a field experiment of a similar intervention in public health facilities in Nigeria and schools in India (Muralidharan and Singh, 2020; Dunsch *et al*., 2023), suggesting that the challenge of improving management practices may generalise beyond our setting.

## 4. Conclusions

There is increasing interest in the role of management practices as a driver of public service delivery in LMICs (Azulai *et al*., 2020; Muralidharan and Singh, 2020; Dunsch *et al*., 2023). In this article we use novel data on the adoption of management practices in over 220 small to medium-sized private health facilities in Tanzania and link these data to quality of clinical care metrics, assessed using SP and clinical observations. The analysis of these data yields three main findings. First, there is considerable variation across health facilities in the adoption of modern management practices, but, overall, management tends to be poor. Second, we find that better managed health facilities are more likely to provide correct treatment in accordance with national treatment guidelines, complete more checklist items and comply slightly more with IPC practices. The relationship with our key quality metric—correct treatment—is substantial in magnitude and clinically meaningful. Third, exploiting a field experiment of a management intervention, we find no significant effect on our primary measure of management and a small and significant effect on the management score most closely related to the practices targeted by the intervention. These findings suggest that improving management practices in this setting is far from straightforward. At the same time, the quality-management association is sufficiently large and robust to motivate future research testing other novel management interventions, with rigorous measurement of performance outcomes.

## Supplementary Material

uead075_Online_Appendix

## Footnotes

1 These interventions include, among others, pre- and in-service training, clinical audits and job aids such as surgical checklists. There is a large literature on the effectiveness of medical technologies, but this is somewhat distinct from the literature on quality of care that typically focuses on ‘the degree to which health services for individuals and populations increase the likelihood of desired health outcomes and are consistent with current professional knowledge’ (Institute of Medicine (US) Committee on Quality of Health Care in America, 2001).

2 The literature on improving the performance of health facilities in LMICs covers a broad range of interventions, some of which are targeted at the level of the organisation (Montagu and Goodman, 2016; Montagu *et al*., 2016; Rowe *et al*., 2018). The types of intervention include provider payment methods, different purchasing arrangements, social franchising, accreditation and managerial supervision among others. Not surprisingly, effects on quality of care vary by intervention, setting and outcome measurement, making it difficult to draw firm conclusions. Also of note is a study in India that shows that the same doctors provide significantly better care in their private practice compared to when they are working in a public clinic (Das *et al*., 2016).

3 This is consistent with a large body of observational evidence outside of the health sector (Scur *et al*., 2021).

4 Condition-specific quality metrics cannot be defined without knowing a patient's underlying condition. But relying on the diagnosis of a doctor is highly problematic when studies have shown correct diagnosis rates in LMICs are typically below 50% (Kwan *et al*., 2019).

5 The SPs deal with a whole range of factors, including patient case mix, ability to pay, patient preferences and patient knowledge (Currie *et al*., 2011). For a more detailed discussion of the advantages and drawbacks of SPs relative to other sources of data on quality, see Das *et al*. (2016), King *et al*. (2019) and Kwan *et al*. (2019).

6 Some studies in the literature have used the term ‘management consultancy’ to describe similar interventions. We prefer ‘management support’ because this is more consistent with the nature of the intervention, how it was characterised in Tanzania and the fact that it was delivered by an NGO rather than a consultancy firm.

7 Dunsch *et al*. (2023) found some effects on the adoption of organisational processes under the direct control of facility staff, but these effects faded away one year after the end of the intervention in Nigeria. Muralidharan and Singh (2020) found no significant effects on support to schools, oversight, teacher absenteeism or classroom processes. Azulai *et al*. (2020) found that a training intervention improved organisational culture, but had no significant effect on management practices in the Ghanaian civil service.

8 The private share is much higher in urban areas. In Dar es Salaam, for example, the private sector accounts for 71% of all health care facilities.

9 In addition to these four components, facilities were given the opportunity to apply for commercial loans from local banks. Through the Medical Credit Fund, PharmAccess worked with the banking system to underwrite loans and hence reduce banks’ investment risk and, at the same time, provided technical assistance to health facilities to improve financial management and business planning. However, this component of the intervention was barely implemented during the course of the trial, with only two health facilities taking out a loan under the Medical Credit Fund.

10 The mean follow-up period was 21.2 months for the health facility survey in which we measured management and IPC practices, and 23.8 months for the SP survey.

11 While we achieved high response rates during data collection, we lack the data to show how representative our study sample is of all private facilities, though the sample has a broad geographical reach, containing facilities located in 64 districts, covering 18 of the 22 regions of mainland Tanzania.

12 A value of 0 represents a facility that was given the lowest scoring category on all 13 management practices and 1 indicates that a facility obtained the highest scoring category on all 13 management questions. In robustness checks, we also experiment with factor analysis approaches to generate a summary score and find that the results remain qualitatively similar.

13 For more details, see https://www.safe-care.org/who-we-are/safecare-standards/.

14 A follow-up study to assess the detection rate of SPs is seen as an important step in ensuring the validity of results. Using a structured questionnaire, we telephoned health facilities four weeks after the SP visits, completing interviews with 225 facilities that represented 901 SP visits. We found a detection rate of 5.2%, which is consistent with other SP studies in low- and middle-income countries (King *et al*., 2019).

15 Antibiotic prescribing behaviour is particularly important for public health (Costelloe *et al*., 2010), given increasing concerns about the global threat of antimicrobial resistance (World Health Organization, 2014).

16 A long-standing concern with clinical observations is the Hawthorne effect (Leonard and Masatu, 2010). To minimise such bias, fieldworkers were coached to observe discreetly from the corner of the room, limit any interaction with either provider or patient and not disclose that observations were focused on infection prevention and control practices. We also found that there is no within provider association between IPC compliance and patient visit order number (King *et al*., 2021).

17 When not using SPs, process and outcome measures of quality of care must typically come from data pertaining to real patients. Examples include in-hospital mortality and disease-specific compliance with established clinical guidelines. In the case of mortality, studies commonly use a risk-adjustment method. However, risk adjustment is only as good as the observables with which to adjust mortality and there is evidence to suggest that these adjustments may be inadequate (Finkelstein *et al*., 2017).

18 Another example concerns the influence patients may exert on the care that they receive from doctors. If more knowledgeable, demanding and vocal patients sort to better managed facilities, quality of care may appear better than is actually the case.

19 Of course, the possibility remains that other differences in unobserved characteristics of individual providers drive the relationship between management and quality. However, the skills of the provider are likely to be a strong determinant of clinical quality in the four SP cases we study. Indeed, in models with the full set of controls, we find that the number of medical officers as a proportion of clinical staff is positively associated with correct treatment, item checklist completion, and negatively associated with unnecessary care and antibiotic prescribing.

20 We have 15 provider qualification dummies, which we interact with the indication fixed effects to account for differences in provider qualification by indication. Because of differences in roles, the indications that health providers were exposed to, varied enormously by type of provider. For example, laboratory technicians and assistants were routinely exposed to injection and blood draw safety, while this was rarely the case for medical officers.

21 The trial is registered at the ISRCTN registry: https://doi.org/10.1186/ISRCTN93644888.

22 King *et al*. (2021) estimated treatment effects using a logit model, which explains the minor differences in the results on quality of care.

23 When we convert the measure of management to a *z*-score, the point estimate is 0.308 SDs (*p* = 0.011) in the model with stratum fixed effects only.

24 There is a relevant parallel with the evaluation of management consultancy in Indian textile firms (Bloom *et al*., 2013), in which the study shows a large effect on specific practices that were directly linked to the intervention. The study does not report results for the more generic management practice measure of Bloom and Van Reenan (2007).

25 See King *et al*. (2021) for more detail behind this calculation. The cost of ${\$}$8,000 compares with a median annual revenue of ${\$}$32,365 of health facilities in our sample.

26 For further discussion of possible reasons for the lack of impact, see King *et al*. (2021).

## Notes

**Online Appendix**

**Replication Package**

## Notes

The data and codes for this paper are available on the Journal repository. They were checked for their ability to reproduce the results presented in the paper. The authors were granted an exemption to publish parts of their data because access to these data is restricted. However, the authors provided the Journal with temporary access to the data, which enabled the Journal to run their codes. The codes for the parts subject to exemption are also available on the Journal repository. The restricted access data and these codes were also checked for their ability to reproduce the results presented in the paper. The replication package for this paper is available at the following address: https://doi.org/10.5281/zenodo.8305425.

We are grateful to Nicole Spieker, Aarke de Graaf, Tobias Rinke de Wit, Jishnu Das, seminar participants, three referees and the Editor for helpful comments and advice. We gratefully acknowledge funding from the Health Systems Research Initiative (Medical Research Council, Economic and Social Research Council, Department for International Development, Global Challenges Research Fund and Wellcome Trust; MR/N015061/1) and the International Growth Centre. Ethics approval was obtained from the IRB of the Ifakara Health Institute (04-2016) and the National Institute of Medical Research (IX/2415) in Tanzania, and the London School of Hygiene and Tropical Medicine (10493) in the UK.
