# Supplementary material for: Management Practices and Quality of Care: Evidence from the Private Health Care Sector in Tanzania
Source: Econ J (London). 2023 Sep 18;134(657):436–56. doi: 10.1093/ej/uead075 (PMC10702364; doi:10.1093/ej/uead075)
Supplement: uead075_Online_Appendix [file uead075_online_appendix.pdf]

Management practices and quality of care: Evidence from the private health care sector in Tanzania

Timothy Powell-Jackson, Jessica J C King, Christina Makungu, Matthew Quaife, Catherine Goodman

Online Appendix A: Data

Selection of study facilities

Eligible facilities were dispensaries and health centres which are members of APHFTA (the Association of Private Health Facilities in Tanzania which represents mainly for-profit facilities), and dispensaries, health centres and hospitals which were members of CSSC (the Christian Social Services Commission which represents most mission facilities). Facilities were ineligible if they refused consent, provided specific services only (e.g. mental health or maternity), or were tertiary hospitals. Facilities were recruited from the Northern, Eastern, Central, Southern and Southern Highlands zones of Tanzania (Lake Zone was excluded because SafeCare had been rolled out there prior to study commencement).

The selection of study facilities was based on a sampling frame of 975 potentially eligible private health facilities. With the implementing partners APHFTA and CSSC, we selected a list of 280 potentially eligible facilities for participation in the study. For the CSSC facilities, we selected a random sample of 124 health facilities, stratified by facility type (dispensary, health centre, hospital). For the APHFTA facilities, we were given a list of 156 health facilities that included dispensaries and health centres. Because of the sampling strategy, we do not claim that the study sample is representative of the broader population of health facilities in the study zones. The study facilities are, however, widely dispersed across both urban and rural areas, in 18 of mainland Tanzania's 22 regions.

The partner organisations approached the 280 potentially eligible facilities to confirm eligibility, carry out sensitisation and obtain written informed consent to participate. Of these, 43 declined to participate in the study or were found to be ineligible, such that 237 facilities were recruited at baseline. Study facilities were recruited from Mar 7, 2016, to Nov 30, 2016. By endline, nine facilities had closed down. Specifically, seven were closed permanently, one was undergoing renovations, and one was open but operating illegally without a license so we could not visit. Our main sample thus comprises 228 health facilities.

Overview of surveys

We use data from four principal sources of primary data: 1) health facility survey; 2) clinical observations; 3) standardised patient survey; and 4) SafeCare assessment survey. The first two surveys were conducted during the same one-day visit to each health facility. The standardised patient survey was conducted approximately three months later. The SafeCare assessment was carried out independently of our data collection activities by the implementing partners, involving a one to two day on-site visit to each health facility.

For the health facility survey, clinical observations and patient exit survey, fieldwork teams visited 228 health facilities between 7th February 2018 and 5th April 2018. The health facility survey collected data on the characteristics of the facility, management practices, patient volume, and revenue, and was completed in all 228 health facilities. Clinical observations of provider-patient interactions were used to collect data on health provider compliance with infection prevention and control. Fieldworkers observed at least one provider-patient interaction in 223 facilities (five facilities had no eligible patients) and one indication in 220 facilities (three facilities had interactions with no indications).

SP visits were carried out between 3rd May 2018 and 12th June 2018 in 227 facilities (one facility owned by a private company served only their employees so SPs could not visit undercover). SPs were used to measure process of care. Each facility received the four SP cases. A total of 909 SP visits were done.

The SafeCare assessment survey was done between 12th October 2017 and 3rd December 2018 in 221 facilities (a further eight facilities closed down and one facility re-opened). It

measured compliance with a set of criteria based on international hospital accreditation standards that have been adapted for use in low-resource settings. These data provide us with our alternative measure of management practices. The survey was completed in 221 health facilities.

Management practices

The facility survey management score draws on data from the management module in the health facility survey. This survey involved an interview with the manager in-charge, record review and direct observation. The manager was asked for written informed consent prior to the commencement of the interview.

Of the 237 health facilities recruited into the study in 2016, nine were closed at the time of the health facility survey in 2018.[1] We visited 228 health facilities and successfully completed the health facility survey in all these facilities. We therefore have management data on a sample of 228 health facilities. The health facility survey involved visiting each health facility and remaining on-site for an entire day. Facility visits were scheduled in advance. On the day of the visit, fieldworkers introduced themselves to the manager in charge of the day-to-day running of the facility, presented a permission letter from the government, and obtained informed consent. They then proceeded with a face-to-face interview in which the management questions were asked. Later in the day, after the interview was completed, the same management practices were verified through direct observation of the practice itself (e.g. of a functioning triage system to prioritise patients on arrival at the health facility) or documentation providing evidence of the practice (e.g. existence of a staff appraisal report). Fieldworkers entered the data digitally into tablets using ODK Collect v.1.12.1.

The section on operations contained questions on the adoption of practices directly related to the provision of care. For example, the survey asked “Does the facility have a triage system – a system in which every patient who enters the facility is assessed (their vitals) whether they need to be seen urgently?”, with answers being from “yes” or “no”. The monitoring and targets section asked about the availability and use of various types of information and the nature of target setting. For example, the survey asked “Do you regularly produce a report reviewing patient indicators? How frequently do you produce these reports?,” with response options ranging from “weekly” to “never.” The human resource management section asked questions on staff appraisal and training. For example, “Do you have a training plan in place to improve the skills of your health workers going forward? How many years does this plan cover?,” with response options ranging from “long-term plan (more than one year) in place” to “no training plan in place.” Finally, the financial management section contained simple questions about financial record keeping. For example, “Do you have an annual statement of the facility revenue and expenditure?,” with the response options being “yes” or “no”.

We normalise the responses from the 13 management questions on a 0 to 1 scale. To do so, we rank the response options from best to worst based on those that are more frequent, comprehensive or specific, with the best response receiving 1 and the worst response 0. The 13 management practices are listed in Table A1, with the corresponding score for each response option. For example, in response to the question “does the facility have a triage system?” the category “yes” is assigned a value of 1, and “no” is given 0. Some questions have more response categories. For example, in response to the question “how frequently do you report patient indicators of quality,” the category “weekly” is given 1, “monthly” 0.75, “quarterly” 0.5, “yearly” 0.25, and “never” 0.

---

[1] Of the nine we term as closed, seven were closed permanently, one was undergoing renovations, and one was open but operating illegally without a license so we could not visit.

We then take the unweighted average of the scores from the 13 questions to generate a summary score. This score ranges from 0 to 1, and is interpreted as the proportion of the maximum score obtainable. A value of 0 represents a facility that was given the lowest scoring category on all 13 management practices and 1 indicates that a facility obtained highest scoring category on all 13 management questions.

The second management score draws on data from the SafeCare assessment survey. [2] In 2016, prior to the endline SafeCare management survey, we pre-specified 55 out of the 170 practices that we considered to be management-related. The 55 management practices are listed in Table A2. Each practice was recorded as either non-compliant, partially compliant or fully compliant, aided by a detailed descriptor used to evaluate each practice. We score "non-compliant" as 0, "partially compliant" as 0.5, and "fully compliant" as 1, and take the unweighted average across all the management practices to generate a summary score. As with our preferred measure of management, this score is scaled from 0 to 1 and is interpreted as the proportion of management practices adopted.

Process of care (standardised patients)

SP survey

Standardised patients (SPs) are healthy people, who covertly pose as real patients and respond to the clinician's actions as a real patient would. We used the SPs to measure process quality of care. We developed four SP cases: asthma, non-malarial febrile illness, tuberculosis, and upper respiratory tract infection. Facilities and implementing partners were blinded to the four cases chosen.

We settled on the four SP cases after conducting a comprehensive review of the literature (King, Das et al. 2019) and a systematic assessment of conditions with respect to their clinical significance, disease prevalence, risk to the SP, ethics, and feasibility (see below). The tools and protocols were developed through a number of additional steps. First, we organized a workshop in Dar es Salaam in January 2017. The workshop was attended by the study team, representatives of PharmAccess International and PharmAccess Tanzania, implementing partners for the SafeCare intervention (APHFTA and CSSC), and clinical specialists from Ifakara Health Institute. Second, the study team reviewed the tools and protocols available for two SP studies carried out in India, one examining TB care, the other asthma, angina and childhood diarrhoea. Third, the study team consulted with the expert working group and reviewed the national treatment guidelines for Tanzania to ensure that appropriate history taking, examinations and diagnostic tests would lead a clinician to the correct diagnosis and treatment. Finally, the cases were further refined through repeated piloting with health providers.

For the asthma case, SPs gave the opening statement "I have had a problem with breathing, and last night it became terrible." For the non-malarial febrile illness case, the initial presentation was "I have a fever and I think I have malaria." For the tuberculosis case, the SP started by saying "I have had a cough and it is not getting better." For the upper respiratory tract infection case, the SP gave the opening statement "I have a cough and my head and throat hurt." In each case, the SP gave further details in response to history taking questions (see online Appendix for more details on the SP scripts and protocols).

We recruited 35 fieldworkers for training. Using a combination of intensive role-play in the classroom and clinical visits in the real-world, we trained them over 11 days to consistently portray two cases and accurately recall details of the interaction. We developed a set of strategies to minimise the risk faced by fieldworkers (see below). At the end of the training,

---

[2] For more details see: https://www.safe-care.org/who-we-are/safecare-standards/

we evaluated the participants and selected 17 of the best performing SPs to carry out the fieldwork, of which 47% were female.

Case choice rationale
A shortlist of conditions was drawn up based on (i) a literature review of use of standardised patients in LMICs, and (ii) conditions reported to be frequently treated in facilities in Tanzania. Each condition on the shortlist was then assessed for inclusion on the basis of five criteria:

1. Evidence for treatment: is there clinical evidence (preferably national standard treatment guidelines by which to define correct treatment or management? This was a prerequisite for consideration.
2. Clinical and public health significance: does recognition and correct treatment of the condition have an important public health role, or is it a serious clinical emergency?
3. Frequency in study facilities: is the condition commonly enough seen in study facilities that correct recognition and treatment is feasible, and it will not arouse suspicion?
4. Risk to fieldwork and ethical considerations: will the case necessitate practices which expose the fieldworker to hospital-acquired infection, invasive examinations or a life-changing diagnosis?
5. Falsifiability of symptom and ease of diagnoses: can the symptoms be easily falsified by fieldworkers and will the provider be able to make a diagnosis on the basis of those symptoms during a single consultation with limited laboratory testing?
6. Universal applicability: can the condition be diagnosed or treated, or an appropriate referral made, at all facilities in the study?

The assessment by the study team is summarised in Table A3, which was used to select the four SP cases used in the study.

SP scripts and background stories
The SP fieldworkers were trained over a two week period. The main purpose of the SP training was to teach SPs about the case they are meant to portray, and how they should go about doing this. For each case we developed an SP script, which provided the basis for each fieldworker to learn their role. Table A4 shows the initial presentation for each case and the further details given by the SP if asked. The full SP scripts are available on request. Specifically, SPs were told about the conditions they were acting, and the symptoms the patient would and would not have. SPs were trained to only give information that was asked for by a care provider, and not to deliberately give more information to ‘help’ the provider along the way to a diagnosis. SPs practised coming up with answers for unexpected questions, so that they were prepared to give an answer if a provider asks about a symptom or lifestyle factor that the study team did not anticipate.

Time during training was dedicated to developing background stories for the SP characters. SPs were trained to portray people from a lower middle class demographic group, and to match the type of clients expected at small private providers. SPs were also trained to dress according to this character, and to adapt their dress to different areas of the country where necessary. SPs were given examples of how to explain their attendance at a facility where they are not recognised, and trained to respond to questions about where they are staying and where they are from. The basic backstory, which was adapted according to SP and setting, was that the SP is visiting a relative who has recently been posted to the area for work (for example, the SP’s uncle is a teacher who has been posted to the school). This allowed SPs to explain why they were in the area without requiring them to have local knowledge. SPs worked in groups to develop the ‘personality’ of their SP, working out how their character would respond to different behaviours from providers. This reduced heterogeneity in the portrayal of SP cases across different fieldworkers.

Procedures: consent, data collection, follow-up
We obtained informed consent from health facilities in our sample to receive SPs. They were told that an SP would be visiting their facility unannounced at some point over the next three months but they were given no further details. We sent the four SP cases to each health facility in the sample, randomly allocating fieldworkers to health facilities within each region. At the end of each interaction, SPs completed a debriefing questionnaire on a smartphone using ODK Collect v.1.12.1 immediately after the visit, and fieldwork supervisors verified the information with the SP the same day. The debriefing questionnaire gathered information on the questions, examinations and diagnostic tests completed by the provider as well as the results of these tests, diagnoses offered, and treatment given. The debriefing questionnaires for each SP case are available on request. SPs paid the fees charged and retained medicines and test results to verify information recorded.

To measure SP detection, we telephoned health facilities to interview the manager using a structured questionnaire. This was done four weeks after the SP visits and we completed interviews with 225 facilities that represented 901 SP visits.

Harm minimisation
All fieldwork contains inherent risks, but SP studies expose fieldworkers to additional risks by asking them to pose as real patients. SP cases and training must be designed to minimise these risks. The major risks identified by the study team and other SP manuals, and steps taken to reduce them, are detailed below.

1. Exposure to airborne pathogens in facility. There is little that could be done to reduce exposure of SPs to respiratory pathogens when waiting inside a facility. More serious respiratory infections, such as TB, are not treated in small clinics, and are treated in separate clinics in larger facilities. It was therefore anticipated that the risk of an SP contracting a serious respiratory infection from this work was minimal.
2. Exposure to surface pathogens in facility. During training, SPs were educated about the pathogens that remain on surfaces inside facilities. They were informed of the importance of hand hygiene after the end of the facility visit, and supplied with alcohol hand gel.
3. Exposure to pathogens on thermometers. SPs were trained to avoid having temperature taken orally with an unsterilized oral thermometer.
4. Exposure to pathogens through injections. SPs were trained to avoid all injections, IV fluids and other parenteral administration of medications.
5. Exposure to pathogens through blood draws. SP cases were chosen to minimise likelihood of blood tests, with the exception of the malaria case, which requires a finger-prick blood sample. SPs were trained to avoid having blood drawn except from the fingertip with a single-use, sterile lancet.
6. Unnecessary exposure to ionising radiation. SPs were trained to avoid all X-rays. X-rays were only likely to be offered to TB SPs, and only 37 of 237 study facilities could offer X-ray imaging, so this risk was present only in a small number of cases. If offered, SPs were trained to refuse on grounds of cost.
7. Administration of unnecessary/harmful medications. SPs were trained to avoid ingesting all medications.
8. Invasive physical examinations. SP cases were chosen to minimise the likelihood of invasive physical exams. SPs were trained to refuse pelvic/genital exams and any other examination or procedure they did not feel comfortable with, and to reveal their identity as an SP as a last resort if necessary.
9. Admission to facility. SPs were trained to avoid being admitted to the facility as an inpatient. They refused to be admitted on grounds of inconvenience, saying they needed to return to where they live and seek medical attention there.

10. Diagnosis of previously undetected condition. There was a risk that SPs may be diagnosed with a genuine medical condition during the course of their work, as a direct result of investigations carried out in study facilities. SP cases were designed to minimise this risk. SPs were only recruited if they self-reported good health and did not report any underlying conditions.
11. Abuse or harassment at a facility due to detection of SP. During the first (overt) round of field work, facilities were informed of the use of SPs in the study and asked to give their consent. A letter was given to facilities explaining the use of SPs, to be put on file in the facility along with a copy of the ethical approval. SPs also carried a copy of this letter with them.

Definition of correct treatment and other quality measures

Correct treatment is defined as whether SPs were managed in accordance with the national standard treatment guidelines. The definition of correct treatment for each SP case is shown in Table A4, developed in close consultation with physicians and pharmacists who had expert knowledge of the study context. Correct treatment is a binary outcome. Correct treatment continues to take a value of 1 if unnecessary care is provided alongside correct treatment (see below).

The proportion of checklist items completed is based on information obtained during the SP visit on whether the provider asks appropriate history taking questions and does appropriate examinations. The relevant history taking questions and examinations are condition-specific and based on national treatment guidelines. The outcome is calculated as the number of appropriate history taking questions and examinations completed divided by the total number that should have been completed. This is done separately for each condition – see Table A5 for the details.

Any unnecessary care is a binary outcome and is defined as drugs prescribed for which there is no evidence of treatment effectiveness or palliative benefit, and inappropriate tests ordered. Examples include antibiotics for non-malarial febrile illness, asthma, TB and upper respiratory tract infection. A palliative drug refers to one for which there is evidence that it relieves the symptoms of a condition – e.g. paracetamol for fever in the case of non-malarial febrile illness or TB, cough syrup in the case of TB or upper respiratory tract infection, and xanthines in the case of asthma. To define unnecessary care, all drugs and tests had to be individually coded.

Infection prevention and control (clinical observations)

Clinical observations

Clinical observations of provider-patient interactions were used to collect data on health provider compliance with infection prevention and control. Such practices are vital for patient safety, particularly in the prevention of health care associated infections which are an important cause of morbidity and mortality globally. The assessment was based on the concept of indications – that is, moments in a provider-patient interaction that present an infection risk to either patient, provider or both. For example, if the provider takes the patient’s temperature with a medical thermometer, a patient is exposed to an infection risk. For every indication there is a corresponding action. In the case of a thermometer, a corresponding action is disinfecting the equipment before and after use with rubbing alcohol or bleach. Compliance means the correct action was taken in response to an indication.

We adapted a tool from a study by Bedoya et al (2016), itself based on WHO guidelines, to measure IPC practices according to Tanzania standards. The tool specified 21 indications and corresponding actions across the following five domains of infection prevention and control: hand hygiene, glove use, injection and blood draw safety, disinfection of reusable equipment, and waste management (Table A6). The tool was developed for the purpose of

measuring IPC in the outpatient department of health facilities. It is worth noting that indications and practices observed are nested within provider-patient interactions which are nested within patients which are nested within facilities.

Procedures: eligibility, consent, data collection
Fieldworkers observed provider-patient interactions for six hours in each facility: three hours in the consultation rooms, one and a half hours in the laboratory, and one and a half hours in the injection or dressing room. All patients were eligible if they or their adult caretaker gave verbal informed consent. Individuals below 18 years of age were excluded if they were not accompanied by an adult caretaker

To minimise bias, fieldworkers were coached to observe discreetly from the corner of the room, limit any interaction with either provider or patient, and not disclose that observations were focused on IPC practices. Fieldworkers recorded every indication and action on a paper version of the IPC tool. Providers were asked for written consent prior to commencing observation, but were not shown the tool. The data were double entered in Dar es Salaam on tablets using ODK Collect v.1.12.1.

Fieldwork teams visited 228 health facilities as part of the health facility survey, observing at least one provider-patient interaction in 223 facilities (five facilities had no eligible patients) and one indication in 220 facilities (three facilities had interactions with no indications). In total, 29,608 infection prevention and control indications were observed in 5425 provider-patient interactions[3] involving 2840 patients. There were 1,737 provider-patient interactions in which no indication was triggered, leaving 3,688 provider-patient interactions with at least one indication observed.

Definition of compliance
IPC compliance is defined as whether the correct action is observed in response to an indication (infection risk to patient or provider). The outcome is defined at the level of indication, and hence is binary.

[3] There were 1,737 provider-patient interactions in which no indication was triggered, leaving 3,688 provider-patient interactions with at least one indication observed.

Table A1. Health facility survey management practices

	Management practice question	Coding (score)
1	Does the facility have a triage system – a system in which every patient who enters the facility is assessed (their vitals) whether they need to be seen urgently? Who does this check?	Yes (1)<br>No (0)
2	Do you use patient records? These are files containing the medical history of a patient that do not leave the health facility. Are there patients who you don't create a patient record for? Please explain.	Yes (1)<br>No (0)
3	Do you submit information on patient cases to MTUHA – the government's health management information system?	Yes (1)<br>No (0)
4	Do you monitor any patient indicators at this facility other than those submitted to MTUHA. By patient indicators, I mean numbers which you measure to tell you something about whether the facility is providing quality services. For example, you might monitor the number of women referred to PMTCT. If you do monitor them, how many do you monitor? Can you give examples?	1-2 patient indicators (1/3)<br>3-9 patient indicators (2/3)<br>10+ patient indicators (1)<br>No patient indicators (0)
5	Do you regularly produce a report reviewing these patient indicators? By report, I mean a document in which you have tables or graphs to help your team review the performance of this facility. How frequently do you produce these reports?	Yearly (1/4)<br>Quarterly (1/2)<br>Monthly (3/4)<br>Weekly (1)<br>Never (0)
6	Do you monitor any business performance indicators at this facility? For example, you might monitor the income from NHIF or cash patients. These are numbers which tell you about the business performance of the facility. If you do monitor them, how many do you monitor? Can you give examples?	1-2 business indicators (1/3)<br>3-9 business indicators (2/3)<br>10+ business indicators (1)<br>No business indicators (0)
7	Do you regularly produce a report reviewing these business performance indicators? How frequently do you produce this report?	Yearly (1/4)<br>Quarterly (1/2)<br>Monthly (3/4)<br>Weekly (1)<br>Never (0)
8	Do you set targets for this facility to achieve? By targets, I mean specific numerical or quantitative goals which you aim for the facility to reach in the future. What kind of targets do you set – can you give examples? *Probe over what period of time are the targets set.*	Yes (1)<br>No (0)
9	Do you have a training plan in place to improve the skills of your health workers going forward? *Probe how many years does this plan cover.*	Yes (1)<br>No (0)
10	Do you have any formal system to appraise the performance of health care workers? For example, in the government sector they use an appraisal system called OPRAS.	Yes (1)<br>No (0)
11	Do you keep an inventory of your drug stock? That is, do you physically count how many of each drug you have from time to time? How often do you carry out an inventory of your stock?	Yes (1)<br>No (0)
12	Do you have an annual budget of the likely costs the health facility will face over the next year?	Yes (1)

		No (0)
13	Do you produce an annual statement of the facility revenue and expenditure?<br>*Probe by asking about profit and loss statement*	Yes (1)<br>No (0)

Notes: The table shows the questions as asked to the facility manager during interview. When these same management practices were observed for compliance, these questions were obviously not asked. The coding and scores reflect the response options in the module for the observed management practices. In the module for the self-reported management practices, response options were more detailed for some questions, in which case they were re-coded into more aggregated categories shown in this table.

Table A2. SafeCare assessment management practices

	Management practices
1	The governance structure is documented (organogram/chart).
2	Those responsible for governance define, approve and monitor the health care facility's strategic plans, mission statement, operational plans and policies.
3	The health care facility has a current license, issued by an acknowledged health care licensing authority, to operate as a health care facility.
4	A health care facility leader(s) is appointed and is responsible for operating the health care facility and carrying out the health care facility's mission.
5	The health care facility leader(s) defines the care and services to be provided, compliant with national rules and regulations, and documents this in a service charter.
6	A qualified or experienced individual is designated for supply chain management.
7	The program is directed or managed by a designated individual or quality team.
8	Books of accounts are kept in a manner that is appropriate for the size and complexity of the health care facility and external financial reporting meets national bookkeeping standards.
9	The health care facility has developed and implemented appropriate cash management practices in Standard Operating Procedures.
10	There is a Fixed Asset Register (FAR) which contains the relevant information for all fixed assets in the health care facility.
11	The health care facility leader(s) has defined which processes and services require auditing.
12	A designated, licensed individual directly supervises the activities of the pharmacy or pharmaceutical service.
13	A procurement guideline is available and conforms to country-specific requirements regarding a secure supply chain, and regarding specific agents and preferred/approved suppliers.
14	The main storage area is protected from heat and light and the temperature is monitored and effectively regulated.
15	Medications are labelled with essential information according to national regulations.
16	Prescriptions contain all relevant information according to the national guidelines.
17	Medications dispensed are clearly labelled with the name of the medication, dose, name of the patient, date and instruction for use.
18	Staff is guided in recording and reporting of medication errors or adverse drug reactions.
19	The health care facility keeps records of age, physical condition, and maintenance performed on all (medical) equipment.
20	Cleaning staff is aware of general hygiene, infection control, and safety precautions.
21	Mops and brooms are cleaned and dried before being stored.
22	The health care facility has, and has implemented, standard operating procedures for the safe segregation, collection and transportation of all types of waste.
23	There is a staffing plan based on accepted national or international norms.
24	There is a process to ensure that skills and performance of health care facility staff is at the expected level.
25	Each staff member has a written job description which defines their responsibilities.
26	The information and documents noted in the Standard Intent are documented for each staff member.
27	There is a process for evaluating and verifying the credentials (license, education, training and experience) of nurses and other health professionals (non-physicians) working in the health care facility.
28	New clinical staff members are oriented to the health care facility and to their job responsibilities and any specific assignments.
29	All staff are provided with on-going in-service education/training.
30	There is a policy that guides the process of gaining and recording informed consent from the patient, including for which procedures or treatments this is required.
31	There is a process to allow complaints to be heard.
32	The opening hours of the health care facility are displayed and compliant with country regulations.
33	The health care facility has a health information management system that contains the data collected about provision of health services and management of the health care facility.
34	There are regular scheduled meetings, to identify the most frequently diagnosed diseases and morbidities.
35	Each patient has a health record which has a unique identifier number.
36	Policies and procedures on infection prevention control (IPC) are in place and guide the staff in the implementation.
37	Health care waste collection assets are available and allow for color-coded segregation.
38	The lay-out of the health care facility allows for effective flow of patients.
39	Adequate PPE is available for staff (gloves, gowns, etc.) and staff can explain how and when to use these.
40	Staff wrap, handle and store sterile packs according to guidelines.
41	The triage process is guided by documents such as checklists/protocols/guidelines.
42	The health care facility has prepared a list of appropriate referral facilities for patients in need of (specialized) services not provided at the health care facility.
43	A duty roster for the relevant caregivers, including weekends and public holidays and after hours is available and known by the inpatient staff members.

44	Regular ward rounds lead to an appropriate re-assessment of patients and an update of the care plan, and both are documented.
45	There is 24-hour access for staff to pharmaceuticals within the health care facility.
46	There is a guideline for the handling and processing of contaminated materials and infectious waste.
47	Staff is aware of correct wrapping, handling and checking sterility of packs.
48	A designated qualified staff member is responsible for managing the laboratory.
49	The lay-out of the laboratory service is in line with the in-country regulations.
50	There are guiding documents for the labelling of specimens throughout the specimen processing activities and these guidelines are followed.
51	There is an SOP for each assay/test performed in the laboratory.
52	All reagents are stored and labelled according to manufacturers' instructions/directives or guiding document.
53	Internal quality controls (IQC) are performed and recorded for each assay/test to verify reagent/kit quality.
54	Results are registered in a logbook in an orderly manner.
55	A referral register for the referred specimens is kept.

Notes: The response options to each item are: fully compliant; partially compliant; not compliant. These response options are aided by a detailed descriptor used to evaluate each practice (not shown). In scoring the response options we assigned a value of 1 for "fully compliant", a value of 0.5 for "partially compliant" and a value of 0 for "not compliant".

Table A3. SP case choice criteria

Case/condition	Clinical and public health significance	Frequency in study facilities[1]	Risk to fieldworker and ethical considerations	Falsifiability of symptoms and ease of diagnosis	Universal applicability
Included:					
Asthma	Some (not infectious, can be life-threatening)	Low (40/234)	Low- blood tests only to exclude other conditions	Good- can report distinctive breathing difficulties	Yes
Malaria	High (life-threatening, infectious, resistance)	High (221/234)	Some- reduced risk with fingerprick testing with single-use lancets	Good- cyclic pattern of fever means no fever required at consultation	Yes
TB	High (underdiagnosed, infectious)	Low (assumed)	Low- X-ray required but not in facility	Good- history of cough and weight-loss, cough need not produce blood	Yes
Upper respiratory tract infection	High (antimicrobial stewardship)	High (178/234)	Low- blood tests only to exclude other conditions	Good- generic symptoms of headache, coughing and running nose	Yes
Excluded:					
Angina	Limited (life-threatening, not infectious)	Low (assumed)	Low- blood tests only to exclude other conditions	Limited- angina patients typically appear seriously unwell	Yes
Child (any condition, absent)	High (often infectious, significant morbidity)	High (assumed)	Low- child is absent	Poor- attending health facility without child abnormal	Yes
Child (any condition, present)			High- child SPs cannot give consent to study participation	Limited- would need to train children	Yes
Depression	High (significant morbidity, underdiagnosed)	Low (assumed)	Low- blood tests unlikely	Limited- unlikely to be recognised in non-specialist facilities	Yes
Diabetes	High (significant morbidity, underdiagnosed)	Low (65/234)	Some- blood glucose test requires fingerprick	Limited – symptoms can be falsified but not blood glucose levels	Yes
Diarrhoea	High (significant morbidity, infectious)	High (188/234)	Low- blood tests only to exclude other conditions	Poor- can’t provide stool sample	Yes
Family planning client	High	Variable (up to 480 visits per month)[2]	Some- pelvic exam can be refused	Good- no symptoms needed	No[2]
HIV testing	High (significant morbidity, infectious)	Medium (85/234)	High -could be mitigated by testing fieldworkers before study	Good- no symptoms needed	Yes
Hypertension	High (significant morbidity)	Medium (100/234)	Low-blood tests only to exclude other conditions	Poor- cannot falsify high blood pressure	Yes
Injuries and accidents	High (significant morbidity)	Medium (81/234)	Low- blood tests unlikely	Poor- difficult to falsify injuries	Yes

Case/condition	Clinical and public health significance	Frequency in study facilities[1]	Risk to fieldworker and ethical considerations	Falsifiability of symptoms and ease of diagnosis	Universal applicability
Pregnancy testing	Limited (interest in antenatal care, not pregnancy testing)	High (191/234)	Low- blood tests unlikely	Limited- symptoms easily falsifiable but urinalysis will be negative	Yes
Skin diseases	Limited	High (123/234)	Low- blood tests unlikely	Poor- difficult to falsify skin complaints	Yes
Sexually transmitted illness	High (significant burden, infectious)	High (147/234)	Some- pelvic/genital exam, difficult to refuse	Limited- can report pain and discharge but can't falsify visible symptoms	Yes
Urinary tract infection	Limited	High (227/234)	Low- blood tests unlikely	Good- painful and frequent urination	Yes
Worms	High (significant burden)	High (147/234)	Low- blood tests unlikely	Poor- can't provide stool sample	Yes

[1]From situational analysis (SA+) form. Facilities can choose up to ten conditions from a predefined list as the ones most commonly diagnosed or treated. Frequencies listed are the number of facilities which list a given condition as one of their 'top ten'. Data are available for 234 of 237 study facilities.
[2]92 facilities reported a non-zero number family planning clients per month (averaged over the last six months) on the SA+. 60 reported zero clients, and 83 that the question was not applicable. Data are available for 235 of 237 study facilities.

Table A4. SP case description and definition of correct treatment

Case	Initial presentation	Further details given if asked	Correct treatment
Asthma	"I have had a problem with breathing, and last night it became terrible"	Attacks of shortness of breath and wheezing, triggered by exertion, normally at night, lasting 15 minutes to two hours and becoming more frequent over last year	Prescription of inhaled bronchodilator or steroid
Non-malarial febrile illness	"I have a fever and I think I have malaria"	Fever and headache lasting three days, joint and muscle pain. Has taken paracetamol for two days.	Malaria test with a negative result, and no antimalarial prescribed or dispensed
Tuberculosis	"I have had a cough and it is not getting better"	Three week cough with yellow sputum, no blood, low grade fever, chest pain, night sweats, loss of appetite and weight. Completed week course of amoxicillin with no improvement.	Referral for sputum test or to a higher level facility
Upper respiratory tract infection	"I have a cough and my head and throat hurt"	Symptoms for three days, blocked nose and sneezing, no fever.	No antibiotic prescribed or dispensed

Notes: The table summarises the four standardised patient cases which were selected for this study as a measure of quality of care. It shows the statement with which SPs initially presented to providers, further case-specific information which was provided when probed. It also shows our definition of correct treatment for each case based on national standard treatment guidelines. The four cases were chosen after a comprehensive review of the literature (King, Das et al. 2019), assessing the clinical significance and disease prevalence in Tanzania, the risk to the SP enumerator, feasibility, and ethical concerns (e.g. not taking up too much provider time or resources). SP presentations were developed in partnership with an expert working group to make sure that the presentation could lead to correct diagnosis and treatment, and the exact wording and presentation style was refined during piloting and training.

Table A5. SP checklist items (history taking and exams) by case

Asthma (34 items)	Malaria (18 items)	TB (29 items)	Upper respiratory tract infection (20 items)
• Probes time of day of symptoms<br>• Asks if has any allergies<br>• Probes other health-seeking or medication taken<br>• Probes wheezing<br>• Probes chest pain<br>• Asks age<br>• Asks about family history of asthma<br>• Probes type of breathing difficulty (current episode)<br>• Probes circumstances of episode<br>• Probes length of attack<br>• Asks if shortness of breath is constant or episodic<br>• Probes if had eaten anything unusual<br>• Probes previous breathing difficulties<br>• Probes when difficulties started or how long they've happened for<br>• Probes frequency of attacks (how often)<br>• Probes what brings on attacks/if any trigger<br>• Probes if anything improves symptoms/ if you do anything to cope with it<br>• Does the breathing trouble/wake you at night?<br>• How far can you walk during an attack?<br>• Are you breathless even at rest during an attack?<br>• Have your lips become blue during at attack?<br>• Asks if asthmatic<br>• Asks about childhood history of breathing difficulties<br>• Asks occupation/job<br>• Asks if smokes<br>• Probes weight loss<br>• Probes night sweats<br>• Probes fever<br>• Asks if cough produces mucus/sputum<br>• Throat/tonsil exam<br>• Pulse measured<br>• Temperature taken (thermometer, any type)<br>• Blood pressure measured<br>• Listened with stethoscope	• Probes time of day of symptoms<br>• Probes duration of symptoms<br>• Probes other health-seeking or medication taken<br>• Probes duration taking medication<br>• Probes cough<br>• Probes fainting or convulsions<br>• Asks if taken a malaria test<br>• Probes loss of appetite<br>• Probes breathing difficulty<br>• Probes vomiting and/or diarrhoea<br>• Asks age<br>• Asks occupation/job<br>• Asks if smokes<br>• Throat/tonsil exam<br>• Pulse measured<br>• Temperature taken (thermometer, any type)<br>• Blood pressure measured<br>• Listened with stethoscope	• Asks occupation/job<br>• Probes duration of symptoms<br>• Have you had contact with anyone with TB?<br>• Asks if cough produces mucus/sputum<br>• Asks if blood in sputum<br>• Probes weight loss<br>• Probes night sweats<br>• Probes chest pain<br>• Probes fever<br>• Probes time of day of symptoms<br>• Probes loss of appetite<br>• Probes personal history of TB<br>• Asks about family history of TB<br>• Probes wheezing<br>• Asks about family history of persistent cough<br>• Probes breathing difficulty<br>• Asks if smokes<br>• Asks if drinks alcohol<br>• Probes other health-seeking or medication taken<br>• Asks age<br>• Probes personal history of diabetes<br>• Probes HIV testing/status<br>• Probes name or type of medication<br>• Probes duration taking medication<br>• Throat/tonsil exam<br>• Pulse measured<br>• Temperature taken (thermometer, any type)<br>• Blood pressure measured<br>• Listened with stethoscope	• Probes time of day of symptoms<br>• Probes duration of symptoms<br>• Probes fever<br>• Asks if blood in sputum<br>• Probes wheezing<br>• Probes breathing difficulty<br>• Probes other health-seeking or medication taken<br>• Asks if has any allergies<br>• Asks age<br>• Asks occupation/job<br>• Asks if smokes<br>• Asks if cough produces mucus/sputum<br>• Probes chest pain<br>• Probes loss of appetite<br>• Probes personal history of TB<br>• Throat/tonsil exam<br>• Pulse measured<br>• Temperature taken (thermometer, any type)<br>• Blood pressure measured<br>• Listened with stethoscope

Notes: The checklist items are listed in no particular order and there was no requirement that providers should complete items in any particular sequence.

Table A6. IPC indications and actions for compliance

Domain	Indication	Action for compliance
Hand hygiene	Before touching a patient	Provider washed hands with soap or used alcohol hand rub and did not dry hands on reused towel or clothes
	After touching a patient	
	Before a clean/aseptic procedure	
	After exposure to body fluids	
	Before injection or blood draw	
	After injection or blood draw	
Glove use	When using gloves	New gloves were used for each patient
	When carrying out intravenous injection, blood draw, wound cleaning or dressing	Gloves used
	For any other contact with body fluid, mucous membranes or non-intact skin	
	After using gloves	Gloves discarded into waste bin
Injection and blood draw safety	For each injection or blood draw	New sharp used
	For each injection or blood draw	Patient skin prepared with clean swab
	For each injection or blood draw	No needle separation, two-handed re-capping or sharps injury
	For each injection or blood draw	Work surface was clean of infectious waste
Disinfection of reusable equipment	Before or after use of non-infra-red thermometer	Disinfected using rubbing alcohol or bleach
	Before or after use of non-disposable tongue depressor	
	Before or after use of otoscope	
	Before or after use of stethoscope	
Waste management	After use of a sharp	Sharp segregated into safety/improvised sharps container
	After injection or blood draw which produced infectious waste	Swabs, cotton wool, test strips and capillary tubes segregated into red or yellow waste bin with matching bag, or safety/improvised sharps container
	After a medical exam or procedure which produced infectious waste	Swabs, gauze, cotton wool and disposal tongue depressors segregated into red or yellow waste bin with matching bag

Notes: The table summarises the 21 infection prevention and control (IPC) indications that observers were instructed to record at each observation. There are five domains across which indications were recorded: hand hygiene, glove use, injection and blood draw safety, disinfection of reusable equipment, and waste management. Compliant actions were different for each domain, and for most indications within domains except hand hygiene where the action for compliance was consistent across indications.

Online Appendix B: Additional results

Table B1. Summary statistics on additional facility characteristics

Variable	Mean	SD	Min	Max	Facilities	Obs
Manager in-charge						
Clinician (%)	0.70	0.46	0	1	228	228
Administrator (%)	0.08	0.27	0	1	228	228
Both clinician and administrator (%)	0.22	0.41	0	1	228	228
Number of medical doctors as proportion of clinical staff	0.08	0.18	0	1	228	228
Number of outpatient consulting rooms	2.3	2.2	1	24	228	228
Number of inpatient admission beds	28.4	52.6	0	320	228	228

Notes: The table provides summary statistics on additional characteristics of the facility sample.

Table B2. Management and correct treatment by standardised patient case

	(1)	(2)	(3)	(4)
Sample	Asthma	Non-malarial febrile illness	TB	URTI
Dependent variable	Correct treatment	Correct treatment	Correct treatment	Correct treatment
Management score	-0.025	0.43**	0.49**	0.30**
	(0.091)	(0.18)	(0.20)	(0.15)
General controls	Yes	Yes	Yes	Yes
SP fixed effects	Yes	Yes	Yes	Yes
Facilities	227	227	227	227
Observations	227	228	227	227
R-squared	0.124	0.155	0.207	0.200

Notes: Table reports OLS coefficients with standard errors in parentheses. The management score is from the facility survey and is the unweighted average of the score on each of the 13 management questions. Observations are at the SP visit level. The sample in each regression is limited to one of the four cases, an indicated. General controls capture characteristics: facility type, region, profit status, urban location and SafeCare trial arm. SP fixed effects are a dummy variable for each of the SP fieldworkers. *** significant at 1%, ** at 5%, * at 10%

Table B3. Management and prices charged to standardised patients

	(1)	(2)	(3)	(4)
Sample	Full	Full	Full	Control
Dependent variable	Prices charged (US$)	Prices charged (US$)	Prices charged (US$)	Prices charged (US$)
Management score	1.235	0.605	0.534	1.271
	(0.967)	(1.039)	(1.030)	(1.381)
General controls	No	Yes	Yes	Yes
SP and case fixed effects	No	No	Yes	Yes
Facilities	227	227	227	116
Observations	909	909	909	465
R-squared	0.004	0.242	0.296	0.458

Notes: Table reports OLS coefficients with standard errors in parentheses, clustered at facility level. The dependent variable is the amount paid by the standardised patient at each visit (expressed in US$ using an exchange rate of $1 = 2,264 Tsh). The management score is from the facility survey and is the unweighted average of the score on each of the 13 management questions. Observations are at the SP visit level. The full sample is used in all regressions, with the exception of column 4 in which the sample is limited to facilities assigned to the control arm of the SafeCare field experiment. General controls capture characteristics: facility type, region, profit status, urban location and SafeCare trial arm. SP fixed effects are a dummy variable for each of the SP fieldworkers. Case fixed effects are a dummy for each of the SP case presentations. *** significant at 1%, ** at 5%, * at 10%.

Table B4. Robustness to controlling for staff qualifications: management and quality for SPs

	(1)	(2)	(3)	(4)	(5)
Sample	Full	Full	Full	Full	Full
Dependent variable	Correct treatment	Proportion of checklist items completed	IRT checklist score	Any unnecessary care	Number of antibiotics prescribed
Management score	0.252***	0.082**	0.634***	0.018	-0.204
	(0.069)	(0.038)	(0.218)	(0.078)	(0.135)
General controls	Yes	Yes	Yes	Yes	Yes
SP and case fixed effects	Yes	Yes	Yes	Yes	Yes
Staff qualification control	Yes	Yes	Yes	Yes	Yes
Facilities	227	227	227	227	227
Observations	909	908	908	909	909
R-squared	0.402	0.241	0.231	0.165	0.218
Bias adjusted coefficient	0.207	0.049	0.442	0.067	-0.135

Notes: Table reports OLS coefficients with standard errors in parentheses, clustered at facility level. The management score is from the facility survey and is the unweighted average of the score on each of the 13 management questions. Observations are at the SP visit level. The full sample is used in all regressions. General controls capture characteristics: facility type, region, profit status, urban location and SafeCare trial arm. SP fixed effects are a dummy variable for each of the SP fieldworkers. Case fixed effects are a dummy for each of the SP case presentations. The control for staff qualifications is the number of medical officers as a proportion of the clinical staff. The bias adjusted coefficient is the value of β produced when $\delta = 1$ and $R_{max} = 1.3\tilde{R}$. *** significant at 1%, ** at 5%, * at 10%.

Table B5. Robustness to controlling for staff qualifications: management and IPC compliance

	(1)	(2)
Sample	Full	Control
Dependent variable	IPC compliance	IPC compliance
Management score	0.054*	0.087**
	(0.032)	(0.041)
General controls	Yes	Yes
Patient controls	Yes	Yes
Indication x staff qualification fixed effects	Yes	Yes
Facilities	220	114
Observations	29,553	15,234
R-squared	0.682	0.689
Bias adjusted coefficient	0.034	0.067

Notes: Table reports OLS coefficients with standard errors in parentheses, clustered at facility level. The dependent variable is compliance with infection prevention and control practices in all regressions. Observations are at the level of IPC indication. The management score is from the facility survey and is the unweighted average of the score on each of the 13 management questions. The full sample is used in column 1, while the sample is limited to facilities assigned to the control arm of the SafeCare field experiment in column 2. General controls capture characteristics: facility type, region, profit status, urban location and SafeCare trial arm. Patient controls are age and gender of the patient observed. Indication fixed effects (a dummy variable for each IPC indication) are interacted with staff qualification fixed effects (a dummy for each qualification of the health worker observed). The bias adjusted coefficient is the value of β produced when $\delta = 1$ and $R_{max} = 1.3\tilde{R}$. *** significant at 1%, ** at 5%, * at 10%.

Table B6. Robustness checks: management and correct treatment

		Coefficient	R^2	Observations
1.	Baseline	0.29***	0.398	909
		(0.073)		
2.	Additional controls	0.28***	0.399	909
		(0.076)		
3.	Exclude "detected" SPs	0.29***	0.402	861
		(0.075)		
4.	Weight by patient volume	0.28***	0.445	909
		(0.093)		
5.	Probit regression	0.28***	0.362	909
		(0.068)		
6.	Management score (factor analysis)	0.049***	0.397	909
		(0.013)		
7.	Management score (z-score)	0.048***	0.397	909
		(0.013)		
8.	Management score (observed and self-reported)	0.37***	0.400	909
		(0.083)		

Notes: The table shows the robustness of the main SP findings to a range of different specifications, variables, and models. OLS coefficients with standard errors in parentheses, clustered at facility level. Model 1 is our baseline regression, as reported in Table 2 (Panel A, column 3). Model 2 controls for additional covariates, including the training of person in charge of facility, number of beds, and number of consultation rooms. Model 3 restricts the sample to SP visits that not detected as being fake in the follow-up detection survey. Model 4 weights the data by patient volume. Model 5 uses a probit regression and reports the coefficient on management as a marginal effect. Model 6 uses the primary factor from factor analysis to generate the summary management score. Model 7 uses a management z-score. Model 8 uses the mean of the observed and the self-reported management score as the explanatory variable. *** significant at 1%, ** at 5%, * at 10%.

Table B7. Robustness checks: management and IPC compliance

		Coefficient	R^2	Observations
1.	Baseline	0.062**	0.640	29,608
		(0.031)		
2.	Additional controls	0.087***	0.641	29,608
		(0.032)		
3.	Probit regression	0.061**	0.569	29,607
		(0.031)		
4.	Management score (factor analysis)	0.010*	0.640	29,608
		(0.005)		
5.	Management score (z-score)	0.011**	0.640	29,608
		(0.005)		
6.	Management score (observed and self-reported)	0.086**	0.640	29,608
		(0.034)		

Notes: The table shows the robustness of the main IPC findings to a range of different specifications, variables, and models. OLS coefficients with standard errors in parentheses, clustered at facility level. Model 1 is our baseline regression, as reported in Table 3 (Panel A, column 4). Model 2 controls for additional covariates, including the training of person in charge of facility, number of beds, number of consultation rooms.. Model 3 uses a probit regression and reports the coefficient on management as a marginal effect. Model 4 uses the primary factor from factor analysis to generate the summary management score. Model 5 uses a management z-score. Model 6 uses the mean of the observed and the self-reported management score as the explanatory variable. *** significant at 1%, ** at 5%, * at 10%.

Table B8. Baseline balance on facility characteristics

	Treatment		Control	
	Mean	SD	Mean	SD
Type of organisation				
For-profit, APHFTA (%)	0.51	0.50	0.50	0.50
Faith-based, CSSC (%)	0.49	0.50	0.50	0.50
Facility type				
Dispensary (%)	0.55	0.50	0.57	0.50
Health Centre (%)	0.30	0.46	0.28	0.45
Hospital (%)	0.15	0.36	0.15	0.36
Facility location				
Inside Dar Es Salaam (%)	0.19	0.39	0.18	0.39
Outside Dar Es Salaam (%)	0.81	0.39	0.82	0.39
Location type				
Urban (%)	0.36	0.48	0.29	0.45
Peri-Urban (%)	0.25	0.43	0.29	0.45
Rural (%)	0.40	0.49	0.43	0.50
Number of outpatient consulting rooms	2.3	2.5	2.3	1.8
Number of inpatient admission beds	30.6	56.0	24.1	47.3

Notes: The table shows summary statistics on baseline characteristics of the sample by treatment group. Treatment sample is 118 facilities. Control sample is 119 facilities.
